# Characteristics and outcomes in children on long-term mechanical ventilation: the experience of a pediatric tertiary center in Rome

**DOI:** 10.1186/s13052-020-0778-8

**Published:** 2020-01-31

**Authors:** Martino Pavone, Elisabetta Verrillo, Alessandro Onofri, Serena Caggiano, Maria Beatrice Chiarini Testa, Renato Cutrera

**Affiliations:** Pediatric Pulmonology & Respiratory Intermediate Care Unit, Sleep and Long Term Ventilation Unit, Academic Department of Pediatrics (DPUO), Pediatric Hospital “Bambino Gesù” Research Institute, Piazza S. Onofrio 4, 00165 Rome, Italy

**Keywords:** Long – term ventilation, Invasive mechanical ventilation, Non-invasive ventilation, Neuromuscular disorders

## Abstract

**Background:**

Children with chronic respiratory failure and/or sleep disordered breathing due to a broad range of diseases may require long-term ventilation to be managed at home. Advances in the use of long-term non-invasive ventilation has progressively leaded to a reduction of the need for invasive mechanical ventilation through tracheostomy. In this study, we sought to characterize a cohort of children using long-term NIV and IMV and to perform an analysis of those children who showed significant changes in ventilatory support management.

**Methods:**

We performed a retrospective cohort study of pediatric (within 18 years old) patients using long-term, NIV and IMV, hospitalized in our center between January 1, 2000 and December 31, 2017. A total of 432 children were included in the study. Long Term Ventilation (LTV) was defined as IMV or NIV, performed on a daily basis, at least 6 h/day, for a period of at least 3 months.

**Results:**

315 (72.9%) received non-invasive ventilation (NIV); 117 (27.1%) received invasive mechanical ventilation (IMV). Children suffered mainly from neuromuscular (30.6%), upper airway (24.8%) and central nervous system diseases (22.7%). Children on IMV were significantly younger when they start LTV [NIV: 6.4 (1.2–12.8) years vs IMV 2.1 (0.8–7.8) years] (*p* < 0.001)]. IMV was likely associated with younger age at starting ventilatory support (aOR 0.9428; *p* = 0.0220), and being a child with home health care (aOR 11.4; *p* < 0.0001). Overtime 39 children improved (9%), 11 children on NIV (3.5%) received tracheostomy; 62 children died (14.3%); and 74 children (17.1%) were lost to follow-up (17.8% on NIV, 15.4% on IMV).

**Conclusions:**

Children on LTV suffered mainly from neuromuscular, upper airways, and central nervous system diseases. Children invasively ventilated usually started support younger and were more severely ills.

## Background

The continuous advances in care and technology, has helped to increase survival of more infants, children, and adolescents with chronic (sometimes critical) conditions. These patients, in some cases, can survive until adulthood and need to be managed with long-term technological support [1–3].

Children with chronic respiratory failure and/or sleep disordered breathing due to a broad range of diseases are part of this group, as they may require long-term ventilation (LTV) to be safely managed at home [1–3].

Congenital or acquired neurological disorders, neuromuscular diseases, complex craniofacial abnormalities, and severe obesity complicated by obstructive sleep apnea are diseases that often require LTV [1, 4].

Previous papers, as single center experiences or multicenter surveys, provided data on the indications and local problems related to the use of LTV. Some studies reported data on children on non-invasive ventilation (NIV), other on children on both NIV and invasive mechanical ventilation (IMV) [4–7].

Advances in the use of long-term NIV has progressively leaded to a reduction of the need for IMV through tracheostomy [1–3]. Information are growing about trends of clinical characteristics, technology used, longitudinal outcomes or discontinuation rates [8, 9]. Furthermore, increasing knowledge is available on how often, when, and which patients with severe or progressive conditions transition from NIV to IMV due to worsening of their underlying condition [8–10].

With this background, we sought to characterize a cohort of children using long-term NIV and IMV and to perform an analysis of those children who showed significant changes in ventilatory support management. Therefore, the objectives of this single-centered study were to: (1) describe the characteristics of children on LTV followed during the 2000–2017 period; and (2) examine the changes in their clinical characteristics, and long-term outcomes including LTV discontinuation and mortality rates.

## Methods

We performed a retrospective cohort study of pediatric (within 18 years old) patients using long-term, NIV and IMV, hospitalized in our center (Pediatric Pulmonology & Respiratory Intermediate Care Unit; Department of Pediatrics, Pediatric Hospital “Bambino Gesù” Research Institute) between January 1, 2000 and December 31, 2017. A total of 432 children were included in the study.

For the purposes of this study, LTV was defined as IMV or NIV, performed on a daily basis, at least 6 h/day, for a period of at least 3 months and carried out mostly in the user’s home or other long-term care facility (outside the hospital).

Basic demographic data (age, sex, place of residence), causes of respiratory failure, type and duration of ventilatory support, and management-related description were collected.

Home health care was provided to children affected by severe and often chronic illness, requiring subspecialist expertise, utilization of and dependence on medical technology such as respiratory support (LTV, chest physiotherapy), devices for nutrition (nasogastric tube, percutaneous endoscopic gastrostomy and/or percutaneous endoscopic jejunostomy, parenteral nutrition), devices for urologic impairment (cystostomy, intermittent catheterization), motor physiotherapy, wheelchair and other equipments.

The Bambino Gesù Children’s Hospital Scientific Board (Rome, Italy) approved the study and parents signed an informed consent.

### Data analysis

Statistical analysis was performed with MedCalc Statistical Software version 18.2.1 (MedCalc Software bvba, Ostend, Belgium; http://www.medcalc.org; 2018). Continuous variables were non-normally distributed, and were summarized as median (interquartile range, IQR), unless otherwise specified. Continuous data were compared by using Mann-Whitney tests. Chi-squared test was used for comparing proportions. A logistic regression model was performed to assess the relationship between types of ventilatory support and patients’ main characteristics. Variables considered in the model included age at starting ventilatory support, major diagnostic categories, receiving home health care assistance and types of ventilator support in order to adjust the analysis for the potential confounding effect. For all analysed parameters, *p* < 0.05 was considered statistically significant.

## Results

### Characteristics of population studied

Four hundred thirty-two children were identified as receiving home LTV on December 31, 2017. Eight patients (1.9%) were resident in the North, 252 (58.3%) in the Centre, 169 (39.1%) in the South, and 3 (0.7%) usually live outside Italy. No differences were observed between the type of ventilation and the area of residence (Table 1).

**Table 1:** Children’s Characteristics and Type of Ventilation

Characteristics	NIV (N° of pts. = 315)	IMV (N° of pts. = 117)	*P* values
Male/Female	165/150	60/57	χ^2^ = 0.041; *p* = 0.8392
Area of residence			
- Northern Italy	8/307	0/117	χ^2^ = 0. 3.02; *p* = 0.08223
- Central Italy	180/135	72/45	χ^2^ = 0.6766; *p* = 0.4108
- Southern Italy	124/191	45/11	χ^2^ = 0.02918; *p* = 0.8644
- Outside Italy	3/312	0/117	χ^2^ = 1.119; *p* = 0.2915
Age at start of LTV (years)	6.4 (1.2–12.8)	2.1 (0.8–7.8)	*p* = 0.0001
Age at December 31, 2017 (years)	12.5 (7–16)	9 (5–15)	*p* = 0.0298
Major diagnostic categories			
- NMDs	99	33	χ^2^ = 0.417; *p* = 0.5185
- Upper airway diseases	105	2	χ^2^ = 45.681; *p* < 0,0001
- Lower airway diseases	28	17	χ^2^ = 2.902; *p* = 0.0884
- CNS diseases	54	44	χ^2^ = 20.323; *p* < 0.0001
- Abnormal ventilatory control	10	8	χ^2^ = 2.860; *p* = 0.0908
- Other	19	13	χ^2^ = 3.202: *p* = 0.0736
Hours/day spent under mechanical ventilation (< 12/≥12)	285/30	49/68	χ^2^ = 114.604, *p* < 0.0001
Home health care (yes/no)	52/263	107/10	χ^2^ = 205.542, *p* < 0.0001

Results are expressed as median (interquartile range), unless otherwise specified

*NIV* Noninvasive Ventilation, *IMV* Invasive Mechanical Ventilation, *NMDs* Neuromuscular Diseases, *CNS diseases* Central Nervous System diseases

The median (interquartile range) age at the start of mechanical ventilation was significantly different in children on NIV or IMV [NIV: 6.4 (1.2–12.8) years or IMV 2.1 (0.8–7.8) years; (*p* = 0.0001)]. The median age of patients still alive on December 31, 2017 was 11.6 (interquartile range 6.0–16.0) years and significantly different for children receiving NIV or IMV [NIV: 12.5 (7.0–16.0) or IMV: 9.0 (5.0–15.0) years; *p* = 0.0298].

The majority of children (315; 72.9%) received NIV (Table 1), delivered by nasal mask (291 (92.4%)). One hundred seventeen children (27.1%) received IMV.

The median duration of home mechanical ventilation was 4.6 (4.0–5.3) years.

**Fig. 1:**
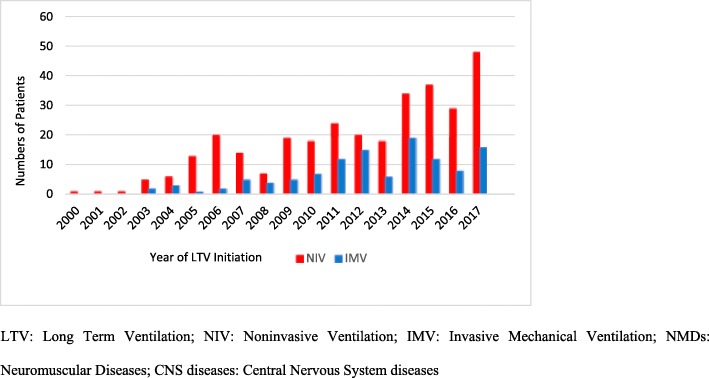
Annual incidence of subjects initiated on long-term mechanical ventilation during the study period

The most frequent underlying condition was neuromuscular diseases (NMDs) (132; 30.6%), followed by upper respiratory airway diseases (107; 24.8%), central nervous system (CNS) diseases (98; 22.7%), lower respiratory airway diseases (45, 10.5%), and abnormal ventilation control diseases (18, 4.2%). A miscellanea of diseases, included in a group of 32 (7.4%) children, was defined “other” (Table 1, Fig. 1).

The most represented neuromuscular diseases were Spinal Muscular Atrophy (SMA) (76 patients, 17.6%), Muscular Dystrophy (28 patients, 6.5%), Congenital Myopathies (19 patients, 4.4%), and Pompe’s Disease (9 patients, 2.1%).

In particular, 44 children were affected by SMA type 1 (32 receiving NIV, 72.7%) and 29 children by SMA type 2 (27 receiving NIV, 93.1%).

The majority of children with upper airway diseases received NIV (105 children; 98.1%). Among these children, 45 (42.8%) presented Obesity; 29 (27.6%) Craniofacial Malformations; 19 (18%) Prader - Willi Syndrome; 6 (5.7%) Laryngomalacia, and 6 (5.7%) Idiopathic Obstructive Sleep Apneas. The remaining 2 children who received IMV presented respectively Pierre-Robin Sequence and Tracheoesophageal Cleft.

Among children affected by lower airway diseases, 28 (62.2%) received NIV and 17 (37.8%) IMV. Children receiving NIV most frequently were affected by Cystic Fibrosis (7; 25%); Tracheobronchomalacia (5; 17.8%), and Bronchopulmonary Dysplasia (3; 10.7%). The majority of children receiving IMV were affected by Bronchopulmonary Dysplasia (7; 41%).

**Fig. 2:**
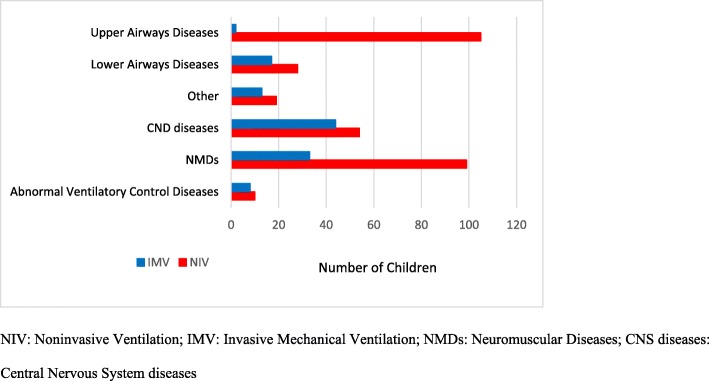
Major Diagnostic Categories and Types of Ventilation

Children with upper airways diseases were more frequently on NIV (χ^2^ = 45.681, *p* < 0.0001). Children with central nervous system diseases were more frequently on IMV (χ^2^ = 20.323; *p* < 0.0001) (Table 1, Fig. 2).

Children on NIV were more often ventilated < 12 h/day (χ^2^ = 111.604; *p* < 0.0001) and received less frequently home health care (χ^2^ = 205.542; *p* < 0.0001) compared to those on IMV. Among children non-invasively treated, 121 (38.4%) received NIV due to sleep disorders of breathing.

Multivariate logistic regression (Table 2) showed that IMV was likely associated with younger age at starting ventilatory support (aOR 0.9428; 95% CI 0.9–1.0; *p* = 0.0220), and being a child receiving home health care assistance (aOR 11.4; 95% CI 6.0–21.5; *p* < 0.0001). Invasive mechanical ventilation was unlikely associated with neuromuscular diseases (aOR 0.13; 95% CI 0.0–0.4; *p* = 0.0010) and upper airways diseases (aOR 0.02 95% 0.0–0.1; *p* = 0.0001).

**Table 2:** Summary of factors potentially influencing the use of invasive mechanical ventilation

Variables	aOR	95% CI		*P* value
		Lower	Upper	
Age at start of LTV	0.9428	0.8964	0.9915	0.0220
NMDs	0.1306	0.0390	0.4380	0.0010
CNS diseases	0.3814	0.1105	1.3158	0.1271
Other diseases	0.3427	0.0870	1.3493	0.1256
Lower airway diseases	0.4283	0.1191	1.5403	0.1941
Upper airway diseases	0.0265	0.0045	0.1575	0.0001
Home Health Care	11.4008	6.0471	21.4944	< 0.0001

*NMDs* Neuromuscular Diseases, *CNS diseases* Central Nervous System diseases

### Long-term outcomes according to the type of ventilation

Children on NIV received overtime more changes in setting compared with those on IMV (NIV: 154/161 patients or IMV: 41/76 patients; χ^2^ = 2.964; *p* = 0.0103). No statistically significant differences have been found comparing the number of children who discontinued LTV due to low compliance/adherence (NIV: 9/306 patients or IMV: 0/117 patients; χ2 = 3.406; *p* = 0.0884).

#### Children who improved

Thirty-nine out of 432 (9%) children overtime improved. Among them, 33 out of 315 children (10.5%) were on NIV; 6 out of 117 children (5%) were on IMV (*p* = 0.851).

All children on NIV who improved, discontinued ventilatory support and switched to spontaneous breathing. Among children on IMV who improved, one child was decannulated and switched to NIV; four children discontinued ventilatory support and were switched to spontaneuous breathing trough tracheostomy; one child discontinued ventilatory support, was decannulated and placed on spontaneous breathing.

Comparison of the characteristic of these children according to the type of ventilation are shown on Table 3. Ages at start of LTV [NIV: 6.4 (12.5–16.4) or IMV: 2.1 (1.1–3.4); *p* = 0.0001] and at December 31, 2017 [NIV: 12.5 (11.0–13.6) or IMV: 9.0 (7.0–11.0); *p* = 0.0298] were significantly different in children on NIV or IMV. Patients on NIV who improved were children with upper respiratory airway diseases (χ^2^ = 11.055; *p* = 0.0009), who spent more often < 12 h/day on ventilatory support (χ^2^ = 3.997; *p* = 0.0456), who requested less frequently home health care (χ^2^ = 8.546; *p* = 0.0035). Patients on IMV who improved were children with lower respiratory airways diseases (χ^2^ = 13.958; *p* = 0.0002) and abnormal ventilatory control diseases (χ^2^ = 5.500; *p* = 0.0190).

**Table 3:** Characteristics of children who improved

Characteristics	NIV	IMV	*p* values
N° of Subjects	33/282	6/111	χ^2^ = 2.964; *p* = 0.0851
Male/female	21/12	3/3	χ^2^ = 0.389, *p* = 0.5330
Age at start of LTV (years)	6.4 (12.5–16.4)	2.1 (1.1–3.4)	*p* = 0.0001
Age at December 31, 2017 (years)	12.5 (11.0–13.6)	9.0 (7.0–11.0)	*p* = 0.0298
Major diagnostic categories			
- NMDs	1/32	0/6	χ^2^ = 0.182; *p* = 0.6698
- Upper airways diseases	24/9	0/6	χ^2^ = 11.055; *p* = 0.0009
- Lower airways diseases	2/31	4/2	χ^2^ = 13.958; *p* = 0.0002
- CNS diseases	4/29	0/6	χ_2_ = 0.790; *p* = 0.3742
- Abnormal ventilatory control diseases	0/33	1/5	χ^2^ = 5.500; *p* = 0.0190
- Other	2/31	1/5	χ^2^ = 0.784; *p* = 0.3760
Hours/day spent under mechanical ventilation (< 12/≥12)	31/2	4/2	χ^2^ = 3.997; *p* = 0.0456
Home health care (yes/no)	2/31	3/3	χ^2^ = 8.546; *p* = 0.0035

Results are expressed as median (interquartile range), unless otherwise specified

*LTV* Long Term Ventilation, *NIV* Noninvasive Ventilation, *IMV* Invasive Mechanical Ventilation, *NMDs* Neuromuscular Diseases, *CNS diseases* Central Nervous System diseases

#### Children who received tracheostomy

Eleven out of 315 (3.5%) children on NIV received overtime tracheostomy (Table 4). Among them, one child with severe upper airways obstruction discontinued ventilatory support and was placed on spontaneous breathing; 10 children (4 with NMDs, 6 with CNS diseases) received tracheostomy and were switched to IMV.

**Table 4:** Details of children who received tracheostomy

Characteristics		Outcomes
N° of Subjects	11	
Male/female	5/6	
Age at start of LTV (years)	3.9 (1.2–8.2)	
Age at Tracheostomy (years)	8.1 (1.7–10.9)	
Major diagnostic categories		
- NMDs	4	IMV after Tracheostomy (1 death)
- Upper airways diseases	1	SB after Tracheostomy
- CNS diseases	6	IMV after Tracheostomy (1 death)

Results are expressed as median (interquartile range), unless otherwise specified

*NMDs* Neuromuscular diseases, *CNS diseases* Central Nervous System diseases, *IMV* Invasive Mechanical Ventilation, *SB* Spontaneous Breathing

#### Children who died

Sixty-two out of 432 (14.3%) children overtime died (Table 5). Among them 45 out of 315 (14.3%) children were on NIV and 17 out of 117 (14.5%) children were on IMV (*p* = 0.9488).

**Table 5:** Characteristics of children who died

Characteristics	NIV	IVM	*P* values
N° of Subjects	45/270	17/100	χ^2^ = 0.00413; *p* = 0.9488
Male/female	16/29	9/8	χ^2^ = 1.525, *p* = 0.2169
Age at start of LTV (years)	2.6 (0.8–9.2)	2.3 (0.8–6.5)	*p* = 0.843566
Major diagnostic categories			
- NMDs	25/20	5/12	χ^2^ = 3.322; *p* = 0.0683
- Upper airway diseases	2/43	0/17	χ^2^ = 0.768; *p* = 0.3808
- Lower airway diseases	7/38	3/14	χ^2^ = 0.0393; *p* = 0.8429
- CNS diseases	7/38	7/10	χ^2^ = 4.558, *p* = 0.0328
- Abnormal ventilatory control	2/43	0/17	χ^2^ = 0.768; *p* = 0.3808
- Other	2/43	2/15	χ^2^ = 1.078; *p* = 0.2992
Hours/day spent under mechanical ventilation (< 12/≥12)	34/11	7/10	χ^2^ = 6.406; *p* = 0.0114
Home health care (yes/no)	16/29	16/1	χ^2^ = 16.671, *p* < 0.0001

*LTV* Long Term Ventilation, *NIV* Noninvasive Ventilation, *IMV* Invasive Mechanical Ventilation, *NMDs* Neuromuscular Diseases, *CNS diseases* Central Nervous System diseases

Comparing children who died according to the type of ventilation, no statistically significant differences were found for gender, and age at starting LTV. Subgroup of children on IMV affected by CNS diseases died more often than those on NIV (χ^2^ = 4.558; *p* = 0.0328). Children on IMV ≥ 12 h (χ^2^ = 6.406; *p* = 0.0114) and those receiving home health care (χ^2^ = 16.671; *p* < 0.0001) died more frequently compared with the others.

#### Children lost to follow up

Seventy-four out of 432 (17.1%) children were lost to follow-up (Table 6). Among these, 56 out 315 (17.8%) children were on NIV and 18 out of 117 (15.4%) were on IMV (*p* = 0.5579).

**Table 6:** Characteristics of children lost to follow up

Characteristics	NIV	IMV	*p* values
N° of Subjects	56/259	18/99	χ^2^ = 0.3434; *p* = 0.5579
Male/Female	27/29	8/10	χ^2^ = 0.0766; *p* = 0.7819
Area of residence			
- Northern Italy	2/54	0/18	0.652; *p* = 0.4195
- Central Italy	30/26	11/8	0.106; *p* = 0.7452
- Southern Italy	22/34	7/11	0.0009; *p* = 0.9762
- Outside Italy	2/54	0/18	0.652; *p* = 0.4195
Age at start of LTV (years)	4.7 (0.7–10.3)	9.0 (2.0–14.2)	*p* = 0.058729
Major diagnostic categories			
- NMDs	16/40	5/13	χ^2^ = 0.00416; *p* = 0.9485
- Upper airway diseases	27/29	0/18	χ^2^ = 13.479; *p* = 0.0002
- Lower airway diseases	5/51	4/14	χ^2^ = 2.223; *p* = 0.1360
- CNS diseases	1/55	7/11	χ^2^ = 19.185; *p* < 0.0001
- Abnormal ventilatory control	1/55	1/17	χ^2^ = 0.726; *p* = 0.3941
- Other	6/50	1/17	χ^2^ = 0.418; *p* = 0.5182
Hours/day spent under mechanical ventilation (< 12/≥12)	50/6	7/11	χ^2^ = 19.287; *p* < 0.0001
Home health care (yes/no)	3/53	18/0	χ^2^ = 59.219; *p* < 0.0001

*LTV* Long Term Ventilation, *NIV* Noninvasive Ventilation, *IMV* Invasive Mechanical Ventilation, *NMDs* Neuromuscular Diseases, *CNS diseases* Central Nervous System diseases

Comparing children lost to follow up according to the type of ventilation, there were no statistically significant differences in terms of number of subjects, gender distribution, area of residence, age at start of ventilation. Children on NIV for upper airway respiratory diseases (χ^2^ = 13.479; *p* = 0.0002), treated on NIV for < 12 h/day (χ^2^ = 19.287; *p* < 0.0001) were lost to follow up more often than the other. Children on IMV affected by CNS diseases (χ^2^ = 19.185; *p* < 0.0001), receiving home health care (χ^2^ = 59.219; *p* < 0.0001) were lost to follow up more often than the others.

### Long-term outcomes according to the presence of home health care assistance

One hundred fifty-nine children received home health care assistance. Children receiving home health care improved less frequently (with home health care 5/154 vs without 34/239 patients; χ2 = 10.579; *p* = 0.0011), and died more often than those without home health care (with home health care 32/127 vs without 30/273 patients; χ2 = 6.808; *p* = 0.0091). No statistically significant differences have been found comparing the number of children lost to follow (with home health care: 21/138 vs without 53/220;; χ2 = 2.72; *p* = 0.0991).

## Discussion

In this study, we evaluated main characteristics and outcomes of children undergoing long-term ventilation due to chronic respiratory failure and/or sleep disorders of breathing in a pediatric tertiary center in Rome (Italy). The majority of our patients were non-invasively ventilated trough nasal masks. Furthermore, our children on invasive mechanical ventilation started ventilatory support very young, usually received ventilation for ≥12 h/day, required home health care assistance and died because of the progression of neurological disorders.

In our single center study, children on LTV suffered mainly from NMDs (30.6%); upper respiratory airway diseases (24.8%) and CNS diseases (22.7%). Children affected by upper airways diseases (33.3%) usually started NIV. Conversely, children affected by CNS diseases (37.6%) were supported by IMV. In previous studies, the reported main reasons for LTV were respiratory tract diseases (18 to 60%), NMDs (15 to 56%), CNS diseases (12 to 46%), and congenital and/or acquired abnormal ventilator control diseases (12 to 25%). Main reasons for IMV were congenital anomalies (43%) [11], CNS diseases (22.5 to 50%) [11–13], abnormal ventilator control diseases (52%) [14]. Main reasons for NIV were NMDs (31 to 73%) [11, 14] and respiratory disorders (36%) [12]. Nathan et al. [15], have reported slightly different results from Kuala Lumpur (Malaysia). Their main reasons for initiating LTV were lower respiratory airways diseases (55% on NIV; 10% on IMV), and spinal cord injury (50% on IMV). Our results are similar to those of many studies performed especially in western, more developed and with high-income countries [8–14, 16–20]. Differences between studies may vary depending on several factors such as the availability of local facilities and skills; differences of inclusion criteria in the main diagnostic categories considered, and collaborations with referral.

Children on IMV started ventilatory support younger than children started on NIV [IMV: 2.1 (0.8–7.8) or NIV: 6.4 (1.2–12.8); *p* = 0.0001]. In our logistic regression, IMV was likely associated with younger age at starting ventilatory support (*p* = 0.0220) and receiving home health care (*p* < 0.00001). Some previous studies confirm our findings [12, 14, 17]. Only Kherani et al. [13], reported no differences comparing the age of initiation of ventilation in the NIV and IMV groups in children less than 1 year. Racca et al. [17], reported that IMV was significantly related to younger age, longer time spent under mechanical ventilation, and neuromuscular disorders or hypoxic (ischemic) encephalopathy. These results highlight that children affected by more severe diseases, need home health care and need more frequently IMV. Furthermore, in these children, the need for IMV appears very often early on life.

Overtime 39 children (9%) improved and discontinued ventilatory support. Children on NIV who improved were more often affected by upper airway diseases, ventilated < 12 h/day, and without home health care assistance. Lower respiratory airways diseases more often affected children on IMV who improved. Previous studies report a discontinuation rate from ventilatory support ranging from 3.6 to 45% [15, 16, 18]. Children who discontinued respiratory support were those showing improvement of the underlying condition [8], such as chronic lung diseases, and upper airway abnormalities [9, 10, 14, 16], or those ventilated < 24 h/day [10]. Conversely, children with NMDs were less likely to come off their ventilator compared to children with airway diseases [15]. These results suggest that discontinuation from LTV is possible in less complex patients, especially in those affected by upper airway diseases and ventilated during the night.

Overtime 11 (3.5%) children on NIV worsened. Among them, one child with very severe upper airways obstruction received tracheostomy and switched to spontaneous breathing, 10 children (4 with NMDs, 6 with CNS diseases) received tracheostomy and switched to IMV. For patients already on NIV, previous studies reported a tracheostomy rate varying from 2.1 to 26.8% [8, 14]. Reasons for tracheostomy were repetitive airway infection/aspirations and acute exacerbation of chronic respiratory failure [11]. Koncicki ML et al. [18], reported that 8.9% of their patients transitioned to IMV mainly because of neurologic (58%) and neuromuscular disorders (39%). These results reinforce the concept that children suffering from neurological and NMDs overtime are more prone to deteriorate and may need tracheostomy/IMV.

In our cohort, overtime 62 (14.3%) children died. Among these, 45 (14.2%) children were on NIV and 17 children (14.5%) were on IMV. Children with CNS diseases on IMV (*p* = 0.0328), children performing IMV for ≥12 h/day (*p* = 0.0114), and children receiving home health care (*p* < 0.0001) died more often than the other did. Previous studies reported a deaths rate on NIV ranging from 2.6 to 17.7% [10, 14], and on IMV from 0 to 40% [14, 15]. Deaths have been reported in hospital [10]; because of progression of the underlying disease [10], especially in children with NMDs [8, 13, 16]; in children affected by cardiac, neurologic [8, 11, 18], and chronic lung diseases [15, 16]. Furthermore, deaths have been reported as consequences of accidental disconnection from the ventilator [10, 15] or decannulation [12]. These results support the concept that, overtime, children on LTV with neurological disorders are among those at increased risk of dead.

In our study, 74 (17.1%) children were lost to follow-up. Among these, 17.8% of children were on NIV and 15.4% were on IMV. Children lost to follow up were those on NIV due to upper airway diseases, those ventilated < 12 h/day and those on IMV affected by CNS diseases and receiving home health care. Data from previous studies report 1 to 3% of children lost to the follow up [8, 10, 12]. Our negative data differ from those of previous studies, reporting a much higher dropout rate. Our analysis highlights that two opposite categories of patients were lost to follow up. On one side, the less complex patients affected mainly by upper airway diseases and ventilated at night. On the other side, more complex patients affected mainly by CNS diseases and receiving home health care assistance. The explanation of these results poses some difficulties as we can only speculate on possible causes. The dropout may be due to several reasons including the progressive patients/families lost of motivation in continuing long term follow up due to less complexity. Other reasons may be the difficulties of recurrent and/or long distance travelling of children with medical complexity with their technologies; the preferences for receiving periodical scheduled home multidisciplinary medical consults and/or assistance according to local facilities. All these considerations bring attention to the importance of personalized and structured follow up programs that can help avoiding the dropout of patients needing long-term ventilatory support.

Our study has a number of limitations. We were unable to provide any further data on the long-term outcomes of children lost to follow up. Among children who died, we are not always able to provide the exact ages and causes of death. These missing data enable us from accurately analyze and compare the survival of children and their reasons for death according to the type of ventilation used.

## Conclusions

This study shows details on a single tertiary pediatric center in Rome (Italy) experience managing with children on long-term ventilation. Children on LTV suffered mainly from neuromuscular diseases, or diseases of upper airways and central nervous system. Children invasively ventilated usually started support younger, spent more hours on ventilation, and were more severely ill. Our results confirm that along with the growing number of children on long-term ventilation, it is increasing their medical complexity and their need for high-quality health care programs able to promote their well-being.

## Data Availability

The datasets used and/or analysed during the current study are available from the corresponding author on reasonable request.

## Abbreviations

CNS diseases: Central Nervous System diseases
IMV: Invasive Mechanical Ventilation
LTV: Long – Term Ventilation
NIV: Non – Invasive Ventilation
NMDs: Neuromuscular Diseases
